# Autophagy inhibitor 3-methyladenine attenuates renal injury in streptozotocin-induced diabetic mice

**DOI:** 10.22038/IJBMS.2024.71378.15518

**Published:** 2024

**Authors:** Haiwen Ren, Mengxin Huang, Liwen Ou, Xuan Deng, Xin Wu, Quan Gong, Benju Liu

**Affiliations:** 1 Department of Clinical Laboratory, Bishan Hospital of Chongqing Medical University, Chongqing 402760, China; 2 Health Science Center, Yangtze University, Jingzhou 434023, China; 3 Department of Immunology, Medical School of Yangtze University, Jingzhou 434023, China; 4 Clinical Molecular Immunology Center, Medical School of Yangtze University, Jingzhou 434023, China; 5 Department of Human Anatomy, Medical School of Yangtze University, Jingzhou 434023, China

**Keywords:** 3-Methyladenine, Autophagy, Diabetes, Diabetic nephropathy, HMGB1/NF-κB signaling - pathway

## Abstract

**Objective(s):**

To investigate whether 3-methyladenine (3-MA) can protect the kidney of streptozotocin (STZ) - induced diabetes mice, and explore its possible mechanism.

**Materials and Methods:**

STZ was used to induce diabetes in C57BL/6J mice. The mice were divided into normal control group (NC), diabetes group (DM), and diabetes+3-MA intervention group (DM+3-MA). Blood glucose, water consumption, and body weight were recorded weekly. At the end of the 6th week of drug treatment, 24-hour urine was collected. Blood and kidneys were collected for PAS staining to evaluate the degree of renal injury. Sirius red staining was used to assess collagen deposition. Blood urea nitrogen (BUN), serum creatinine, and 24-hour urine albumin were used to evaluate renal function. Western blot was used to detect fibrosis-related protein, inflammatory mediators, high mobility group box 1 (HMGB1)/NF-κB signal pathway molecule, vascular endothelial growth factor (VEGF), and podocin, and immunohistochemistry (IHC) was used to detect the expression and localization of autophagy-related protein and fibronectin.

**Results:**

Compared with the kidney of normal control mice, the kidney of diabetes control mice was more pale and hypertrophic. Hyperglycemia induces renal autophagy and activates the HMGB1/NF-κB signal pathway, leading to the increase of inflammatory mediators, extracellular matrix (ECM) deposition, and proteinuria in the kidney. In diabetic mice treated with 3-MA, blood glucose decreased, autophagy and HMGB1/NF-κB signaling pathways in the kidneys were inhibited, and proteinuria, renal hypertrophy, inflammation, and fibrosis were improved.

**Conclusion:**

3-MA can attenuate renal injury in STZ-induced diabetic mice through inhibition of autophagy and HMGB1/NF-κB signaling pathway.

## Introduction

Diabetic nephropathy (DN) is a common cause of glomerulosclerosis and end-stage renal disease (ESRD)(1). In the early stages of DN, proliferation and hypertrophy of glomerular mesangial cells are typical pathological features. High glucose induces proliferation of glomerular mesangial and interstitial cells, and produces a large amount of extracellular matrix (ECM) accumulation in the kidney, giving rise to diffuse or segmental fibrosis of the glomerulus, and ultimately leading to ESRD as the disease progresses (2). 

Like many kidney diseases, DN is characterized by the development of proteinuria, which is caused by podocyte apoptosis and loss of function. The subsequent decline of glomerular filtration rate is related to glomerulosclerosis. Podocyte loss and thickening of the glomerular basement membrane are the main characteristics of diabetes-induced proteinuria (3). Podocin is one of the markers of podocytes, and its expression level can reflect the number of podocytes (3).

Recent evidence from clinical and basic experimental studies suggests that the innate immune response plays a critical role in the pathogenesis and progression of DN through toll-like receptors (TLR) and receptors of advanced glycation endproducts (RAGE)-induced non-infectious inflammatory processes (4-6). High mobility group box 1 (HMGB1) is one of the ligands of TLR4 and RAGE, and its interaction can exert pro-inflammatory effects, which means that HMGB1 is an important target for therapeutic strategies in DN.

Autophagy is a protective intracellular process (7). Autophagy involves multiple molecules, and the orderly activation of each molecule forms a coherent process, which is called autophagic flux (8). Among them, molecules commonly used to monitor this process include LC3, yeast autophagy-related protein (ATG) 6 homolog (beclin1/BECN1), and autophagy adaptor protein P62. LC3 is often used as a monitoring indicator of autophagy induction, which can reflect the inhibition of autophagy induction or autophagosome clearance, but it is not a measure of autophagic flux (9). Beclin1/becn1 is a homologous molecule of yeast ATG6 protein, a component of PI3K complex, and the target of autophagy inhibitor 3-methyladenine (3-MA)(9). P62 plays an auxiliary role in autophagy, targeting and transporting specific markers to the autophagic membrane, and then anchoring the substances to be degraded to the autophagic membrane through LC3. After the formation of autophagic lysosomes, p62 is degraded. Generally, an increase in P62 indicates that autophagosome formation is inhibited or autophagosome clearance is blocked (8). 

3-MA is an inhibitor of early autophagosome formation (10-12). In the past, 3-MA was often used as a tool for studying autophagy. Recently, more and more studies have indicated that 3-MA can inhibit proliferation of interstitial fibroblasts and macrophages through autophagic or non-autophagic pathways, such as NF-κB signaling, TGF-β/Smad signaling, and so on (10, 13-19). Additionally, a recent report indicated that 3-MA can alleviate hyperuricemic nephropathy (19). However, there is no report on the effect of 3-MA in DN. Therefore, we intend to study the impact of 3-MA on kidneys in streptozotocin (STZ)-induced DM mice and its mechanism.

## Materials and Methods

***Antibodies and reagents***

**Figure 1:**
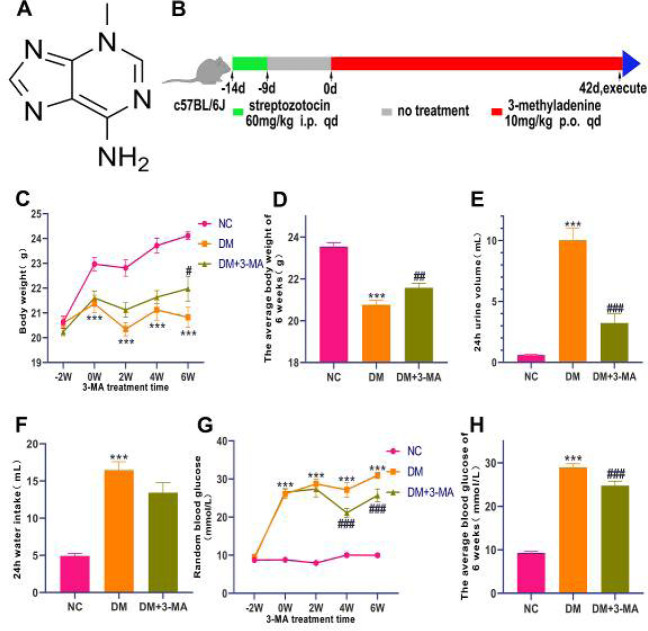
3-Methyladenine (3-MA) reduced blood glucose levels and improved diabetic symptoms in streptozotocin (STZ)-induced diabetic mice

3-MA (molecular structure is shown in Figure 1A) was purchased from Abmole Bioscience (Houston, TX, USA). A bicinchoninic acid (BCA) protein assay kit was purchased from Beyotime (Shanghai, China). Creatinine and blood urea nitrogen (BUN) assay kits were purchased from Nanjing Jiancheng Bioengineering Institute (Nanjing, Jiangsu, China). The Enzyme-linked immunosorbent assay (ELISA) kits for IL-1β and TNF-α were purchased from Elabscience (Wuhan, Hubei, China). The remaining reagent sources are described in our previous research (20).

***Animal experiments***

For this study, animal species and origin, induction of diabetes, and treatment methods are described in our previous research (20). The study was carried out in accordance with the protocols approved by the Institutional Animal Ethics Committee of Yangtze University Health Science Center (approval number: CJYXBEC2019-083).

***24-hour water intake and urine volume***

The 24-hour water intake of mice was recorded every week starting from the first week of 3-MA treatment, the methods of 24-hour urine collection are referred to in our previous research (21). 

***Random blood glucose***

The random blood glucose levels were measured every week. The specific operation method is referred to in our previous research (21).

***Kidney/body weight ratio***

At the end of the experiment, body weight was recorded. Mice were anesthetized and sacrificed, kidneys were rapidly removed and the kidney capsule was stripped. Both kidneys were weighed and the kidney/body weight ratio was calculated.

***24-hour albuminuria assay***

24-hour albuminuria assay is referred to in our previous research (21). In brief, urine albumin was isolated by SDS-PAGE and then measured by Coomassie bright blue staining. 24-hour urinary albumin was calculated by multiplying urinary albumin concentration by urine volume.

***Assessment of renal function and other biochemistry index***

Mice blood samples were collected via enucleation of the eye into the tubes without any anticoagulant to collect the serum. Serum creatinine and BUN were measured using commercially available kits (Nanjing Jiancheng Bioengineering Institute, Nanjing, China).

***Western blotting***

Western blotting is explained to our previous research (20). In brief, the protein was extracted from mouse kidneys after grinding for SDS-PAGE isolation and transferred to the PVDF membrane for chemiluminescence detection. 

***Histology and immunohistochemistry examination***

The kidneys were fixed in 10% neutral formalin liquid for more than 48 hr. Kidneys were cut in half lengthwise, dehydrated in 95% ethanol overnight after ethanol gradient dehydration, then cleared with n-butanol and embedded in paraffin blocks. 4 μm slices were made with a paraffin sectioning machine. After 2 hr of baking, the slices were dewaxed and hydrated, and stained with Sirius Red (Maokang Bio, Shanghai, China) and Periodic acid–Schiff (PAS) staining solution. For IHC staining, the kidney tissue sections were subjected to antigen retrieval with sodium citrate solution, blocked with endogenous peroxidase blocking solution and goat serum, and then incubated with primary antibody. The next day, the kidney tissue sections were incubated with horseradish peroxidase-conjugated secondary antibody for 30 min at 37 ^°^C and then reacted with diaminobenzidine (DAB) to develop color after washing with PBS. Images were acquired using a Leica microscope and the positive area was calculated with Image J software. 

***ELISA analysis***

**The kidney tissues were rinsed with pre-chilled PBS to remove residual blood, and chopped after weighing. The chopped tissue was added to an appropriate volume of PBS containing a 1% protease inhibitor cocktail (usually at a weight-to-volume ratio of 1:9) and triturate well at 4** ^°^ **C using a homogenizer. After ultracentrifugation, the supernatant was recovered for further detection. The levels of IL-1β and TNF-α were examined according to the protocol specified by the manufacturer of the commercial Quantikine ELISA kit (Elabscience, Wuhan, Hubei, China).**

***Statistical analysis***

Data depicted in graphs represent the means±SEM for each group. IBM SPSS Statistics 25 (Version X; IBM, Armonk, NY, USA) was used for statistical analysis. The level of significance between treatment groups was determined using One-way ANOVA and student-*t* multiple comparison tests. *P*<0.05 was considered statistically significant.

## Results

***3-MA reduced blood glucose levels and improved diabetic symptoms in STZ-induced diabetic mice***

It’s well known that the typical clinical manifestations of diabetes are polydipsia, polyphagia, polyuria, and weight loss (22). As shown in Figure 1C-F, DM mice showed less body weight, more water intake, and urinary excretion compared with NC mice; 3-MA-treated DM mice had significantly increased body weight and markedly decreased urinary excretion when compared with DM mice. The results of weekly continuous blood glucose monitoring showed that blood glucose was remarkably reduced in DM mice treated with 3-MA from the 4th week after treatment compared with DM mice, and 3-MA treatment markedly reduced the average blood glucose of 6 weeks in STZ-induced diabetic mice (Figure 1G & H).

***3-MA ameliorated renal hypertrophy and tubule injury in STZ-induced diabetic mice***

**Figure 2:**
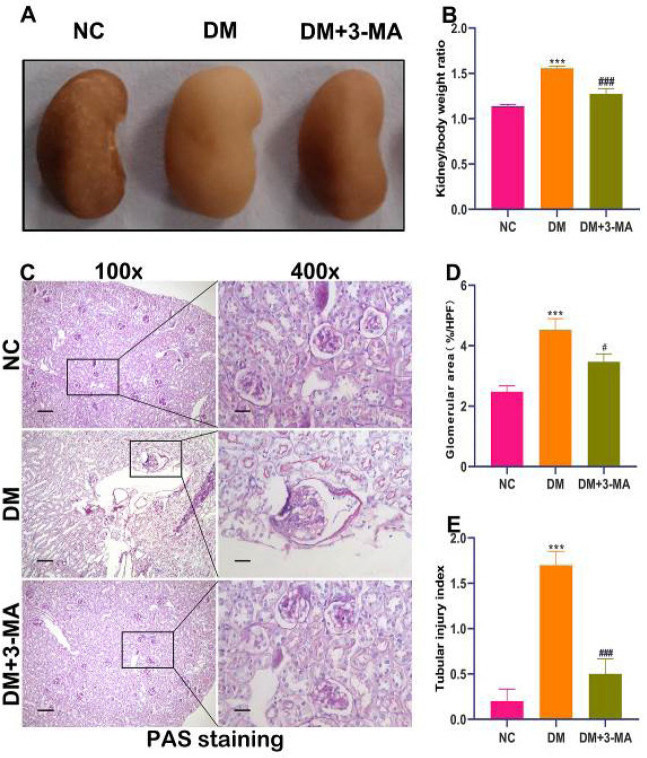
3-Methyladenine (3-MA) ameliorated renal hypertrophy and tubule injury in streptozotocin (STZ)-induced diabetic mice

As shown in Figure 2A, the kidneys of DM mice were paler, more edematous, and hypertrophic in general appearance as compared with the kidneys of NC mice. The upper and lower edges of the kidneys of DM mice were blunt, and the curvature of the left and right edges disappeared. Its general appearance was similar to that of the kidney in membranous nephropathy. However, the general appearance of kidneys in DM mice treated with 3-MA was improved compared with the kidneys of DM mice. After weighing both kidneys of all mice, we found that the kidney/body weight ratio of DM mice was significantly increased compared with NC mice. The kidney/body weight ratio of DM mice treated with 3-MA was remarkably reduced compared with DM mice (Figure 2B). PAS staining further showed severe glomerular hypertrophy and mesangial expansion in the kidneys of DM mice. 3-MA treatment improved the pathology of glomerular hypertrophy, mesangial expansion, and tubular dilation (Figure 2C). Measurement of glomerular area showed that 3-MA treatment markedly reduced glomerular volume in STZ-induced diabetic mice (Figure 2D). Renal tubule space measurements showed that 3-MA treatment markedly reduced the tubular injury index (Figure 2E). The above results suggested that 3-MA ameliorated renal hypertrophy and injury in STZ-induced diabetic mice. 

***3-MA improved glomerular filtration barrier function in STZ-induced diabetic mice***

**Figure 3:**
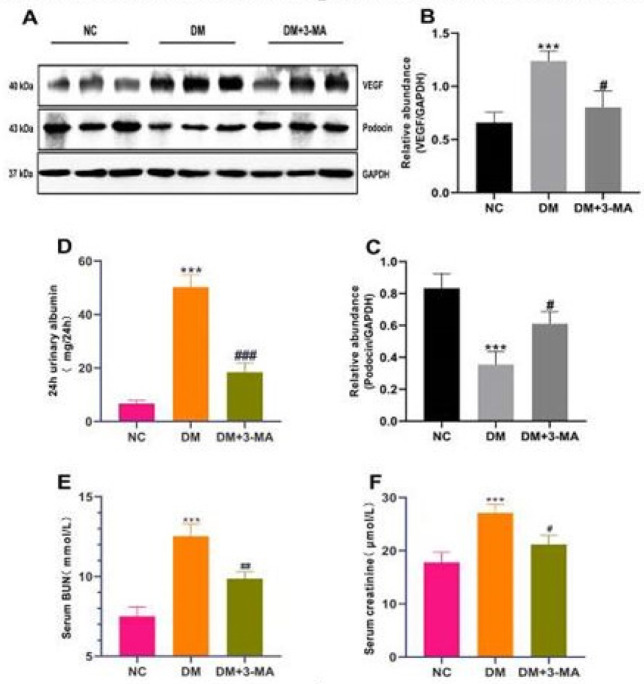
3-Methyladenine (3-MA) improved the glomerular filtration barrier function in streptozotocin (STZ)-induced diabetic mice

It has been reported that hyperglycemia induces podocytes to secrete large amounts of vascular endothelial growth factor (VEGF) when the kidney is exposed to hyperglycemia for a long time. Large amounts of VEGF widen the endothelial space and deform the foot processes, causing massive proteinuria (23). Podocin is a biomarker of the foot process, and its reduced expression can exacerbate proteinuria (24). To investigate whether 3-MA can improve the renal filtration barrier function in diabetic mice, we examined the effect of 3-MA on the expression levels of VEGF and podocin in the kidneys of DM mice. As shown in Figure 3A-C, the expression level of VEGF in the kidneys of DM mice was remarkably up-regulated, and the expression level of podocin was markedly decreased compared with NC mice. While 3-MA treatment markedly decreased the expression level of VEGF and significantly increased the expression level of podocin in the kidneys of diabetic mice. This evidence indicated that 3-MA remarkably improved the renal filtration barrier function in diabetic mice, which was also demonstrated by the result of 24-hour albuminuria, serum creatinine, and BUN (Figure 3D-F).

***3-MA alleviated renal ECM deposition in STZ-induced diabetic mice***

**Figure 4:**
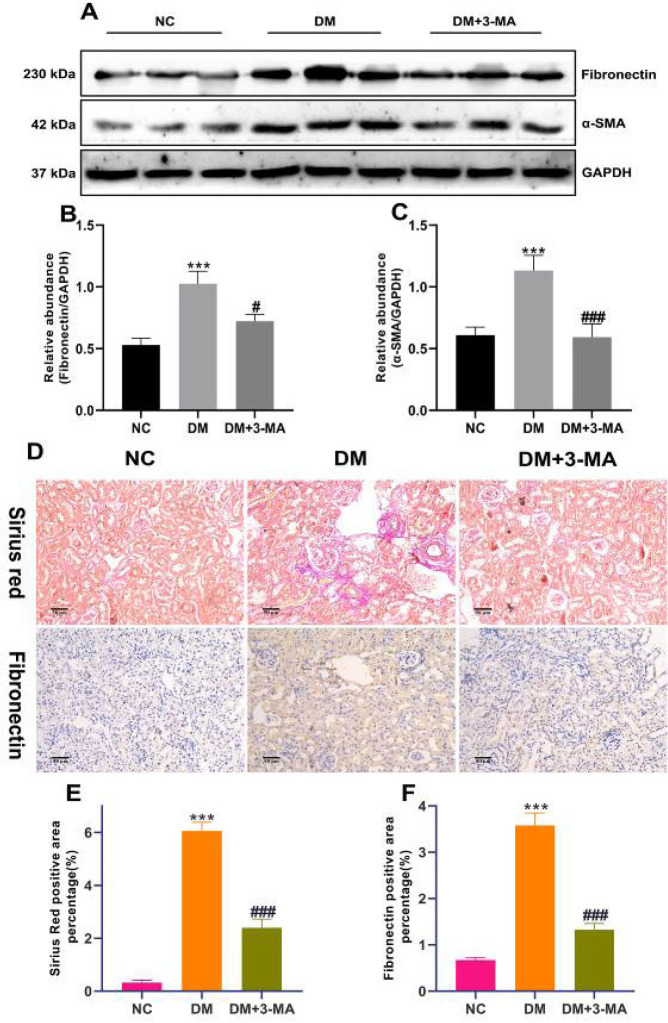
3-Methyladenine (3-MA) alleviated renal ECM deposition in streptozotocin (STZ)-induced diabetic mice

DN is one of the main causes of ESRD, and renal fibrosis plays a crucial role in its pathological development (25). Renal fibrosis is characterized by overexpression and deposition of ECM, which finally leads to scarring of renal tissue (26). Therefore, we explored the effect of 3-MA on the expression levels of renal fibrosis-related markers in STZ-induced diabetic mice and observed the deposition of interstitial collagen in the kidneys. α-SMA is a biomarker after fibroblasts are transformed into myofibroblasts which are the main cells responsible for the production of ECM proteins, and fibronectin is one of the factors that lead to renal fibrosis (25). Therefore, we examined the expression levels of fibronectin and α-SMA by western blotting. As shown in Figure 4A-C, the expression levels of fibronectin and α-SMA were remarkably increased in the kidneys of DM mice as compared with NC mice. While 3-MA treatment significantly reduced the expression levels of fibronectin and α-SMA in the kidneys of diabetic mice. This evidence indicated that 3-MA inhibited ECM protein deposition and fibroblast activation in the kidneys of diabetic mice.

In order to further verify the improvement effect of 3-MA on renal fibrosis in STZ-induced diabetic mice, we performed Sirius Red and IHC staining to observe the deposition of renal interstitial collagen. As shown in Figure 4D, the Sirius Red staining in the kidneys of DM mice showed kenspeckle morphological changes which manifested as mesangial expansion, interstitial expansion, and massive collagen deposition, and the positive area was markedly increased compared with NC mice (Figure 4E). Interstitial dilatation, renal tubular dilatation, and a significant increase in the positive area were also observed in renal fibronectin IHC staining of DM mice, compared with NC mice (Figure 4F). 3-MA treatment clearly improved the renal microscopic morphology of diabetic mice and markedly reduced the positive area of Sirius Red staining and fibronectin IHC staining in the kidneys of diabetic mice (Figure 4D-F). The above evidence indicated that 3-MA significantly reduced renal injury and ECM deposition in diabetic mice.

***3-MA improved renal inflammatory response and inhibited HMGB1/NF-*** ***κB*** ***signaling pathway in STZ-induced diabetic mice***

**Figure 5:**
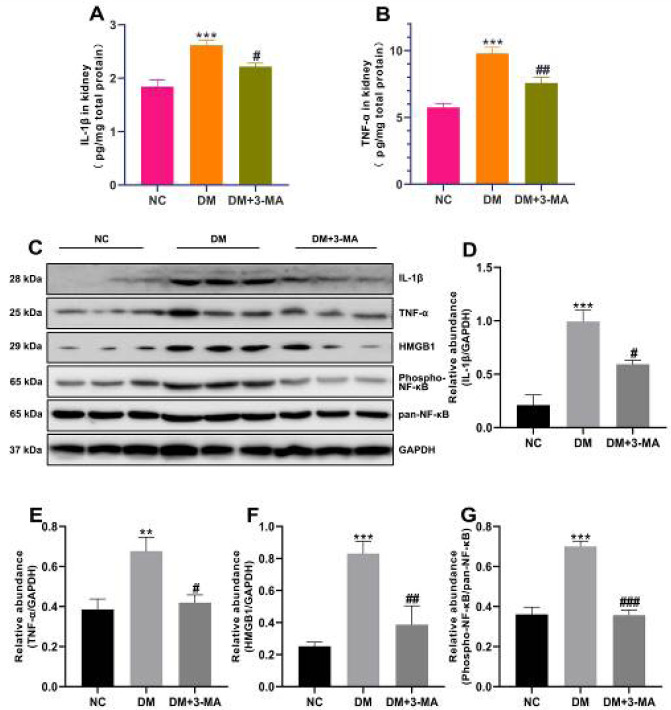
3-Methyladenine (3-MA) improved renal inflammatory response and inhibited HMGB1/NF-κB signaling pathway instreptozotocin (STZ)-induced diabetic mice

Many reports indicate that inflammation is one of the initiating and major factors of DN (27-30). To investigate the effects of 3-MA on renal inflammation in diabetic mice, we used ELISA to detect the expression levels of IL-1β and TNF-α in kidney homogenates. As shown in Figures 5A & B, the expression levels of IL-1β and TNF-α in the kidney homogenates of DM mice were markedly increased compared with NC mice. However, 3-MA treatment significantly reduced the expression levels of IL-1β and TNF-α in the kidneys of diabetic mice. These data indicated that 3-MA ameliorated renal inflammation in diabetic mice.

To further explore the potential mechanism of 3-MA improving renal inflammation in diabetic mice, we detected inflammatory cytokines and related signaling pathway proteins by western blotting. Western blot results showed that the expression levels of IL-1β and TNF-α in the kidneys of DM mice were significantly increased compared with NC mice, and the expression levels of IL-1β and TNF-α in the kidneys of diabetic mice treated with 3-MA were significantly decreased compared with DM mice, which was in line with the results of ELISA (Figure 5C-E). It has been reported that HMGB1 is involved in the process of DN(11, 31-34). HMGB1 activates the downstream protein NF-κB which is the promoter of inflammation-related gene transcription (11, 31-34). As shown in Figure 5C, HMGB1 and phospho-NF-κB were significantly up-regulated in the kidneys of DM mice as compared with NC mice. However, 3-MA treatment inhibited the up-regulation of HMGB1 and phospho-NF-κB in the kidneys of diabetic mice (Figure 5C, F, G). This evidence suggested that 3-MA attenuated the inflammatory response by inhibiting the activation of the HMGB1/NF-κB signaling pathway in the kidneys of diabetic mice.

***3-MA inhibited autophagosome formation in STZ-induced diabetic mice***

**Figure 6:**
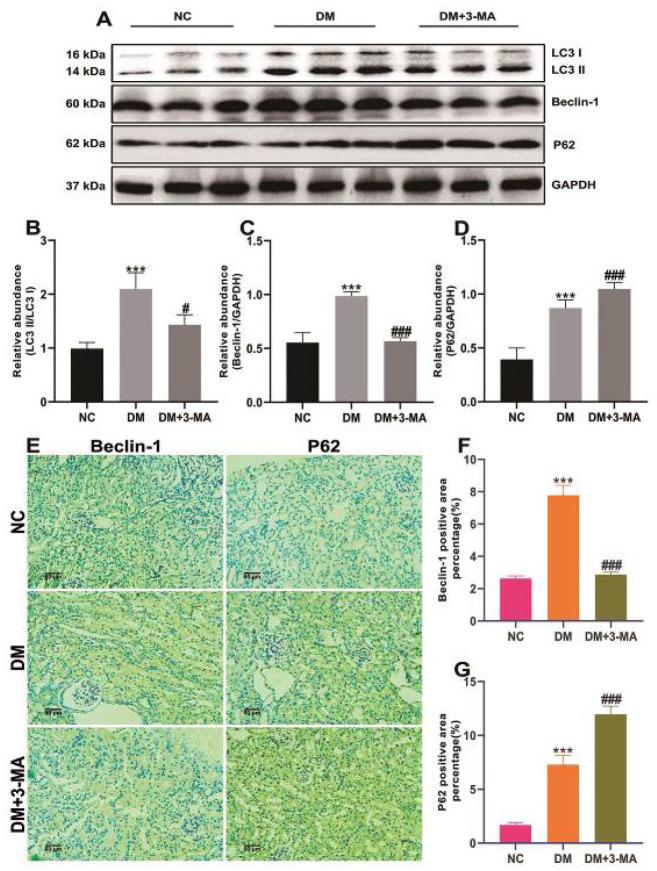
3-Methyladenine (3-MA) inhibited autophagosome formation in instreptozotocin (STZ)-induced diabetic mice

To investigate the protective mechanism of 3-MA on the kidneys of DM mice, we assessed the role of autophagy in DN. As shown in Figure 6A, the results of western blotting of kidney homogenates showed that the ratio of LC3 II/LC3 I, the expression levels of Beclin-1, and P62 in the kidneys of DM mice were remarkably increased compared with NC mice. However, 3-MA treatment markedly decreased the ratio of LC3 II/LC3 I and the expression level of Beclin-1 in the kidneys of diabetic mice, and further increased the expression level of P62 (Figure 6A-D). IHC staining showed the same results (Figure 6E-G). This evidence indicated that autophagy was induced in the kidneys of DM mice accompanied by blocked autophagic flux, and a large number of autophagosomes were retained. 3-MA inhibited autophagy and reduced autophagosome accumulation in the kidneys of diabetic mice.

(A) Chemical structure of 3-MA. (B) Schematic diagram of experimental design for 3-MA treatment of diabetic mice. (C) Weight trend graph over the experimental period. (D) Average body weight for 6 weeks of 3-MA treatment. (E) 24 hr urine excretion. (F) 24 hr average water intake per week. (G) Blood glucose trend graph over the experimental period. (H) Average blood glucose for 6 weeks of 3-MA treatment. ***P<0.001, **P<0.01, and *P<0.05 versus normal control (NC) group. ^###^*P<*0.001, ^##^*P<*0.01, and ^#^*P<*0.05 versus diabetes (DM) group

(A) General appearance of the kidneys. (B) Ratio of kidney weight to body weight. (C) Periodic acid-Schiff (PAS) staining of the kidney tissues. (D) Glomerular area as a percentage of the entire high-power field. (E) Renal tubule injury index. ***P<0.001, **P<0.01, and *P<0.05 versus normal control (NC) group. ^###^*P<*0.001, ^##^*P<*0.01, and ^#^*P<*0.05 versus diabetes (DM) group. All scale bars are 100 μm (100×) or 25 μm (400×)

(A-C) Expression levels of VEGF and podocin in kidneys are determined by immunoblotting. (D) 24 hr urinary albumin quantitative analysis. (E) Expression levels of serum Blood urea nitrogen (BUN). (F) Expression levels of serum creatinine. ***P<0.001, **P<0.01, and *P<0.05 versus normal control (NC) group. ^###^*P<*0.001, ^##^*P<*0.01, and ^#^*P<*0.05 versus diabetes (DM) group

(A-C) Expression levels of fibronectin and α-SMA in kidneys were determined by immunoblotting. (D) Sirius Red staining and the fibronectin immunohistochemistry (IHC) staining in kidneys (200×). (E) Percentage of Sirius red positive area (pink) relative to the whole area. (F) Percentage of fibronectin IHC staining positive area relative to the whole area. ***P<0.001, **P<0.01, and *P<0.05 versus normal control (NC) group. ^###^*P<*0.001, ^##^*P<*0.01, and ^#^*P<*0.05 versus diabetes (DM) group. All scale bars are 50 μm

(A) Expression level of IL-1β in renal homogenate was determined by ELISA. (B) Expression level of TNF-α in renal homogenate was determined by ELISA. (C-G) Expression levels of IL-1β, TNF-α, HMGB1, and phospho-NF-κB were determined by western blotting. ***P<0.001, **P<0.01, and *P<0.05 versus normal control (NC) group. ^###^*P<*0.001, ^##^*P<*0.01, and ^#^*P<*0.05 versus diabetes (DM) group

(A-D) Expression levels of autophagy-related proteins in kidneys determined by western blotting. (E-G) Expression levels of Beclin-1 and P62 in kidneys determined by immunohistochemistry (IHC). ***P<0.001, **P<0.01, and *P<0.05 versus normal control (NC) group. ^###^*P<*0.001, ^##^*P<*0.01, and ^#^*P<*0.05 versus diabetes (DM) group. All scale bars are 50 μm

## Discussion

The global prevalence of DM has been rising steadily. At least half of type 2 DM and one-third of type 1 DM patients develop DN in their lifetime (35). Although current therapeutic strategies have some beneficial effects on the deterioration of renal function associated with DN, none of them are able to prevent the progression of DN. 3-MA is usually used as an autophagy inhibitor for research. Recently, more and more studies are reporting that 3-MA exerts its therapeutic effect on animal models of various diseases, and there is no report of its side effects at present. In this study, we found that the autophagy inhibitor 3-MA can attenuate renal injury in STZ-induced diabetic mice through multiple pathways including autophagy and non-autophagy.

It has been reported that hyperglycemia increases the production of AGEs and ROS, which leads to increased expression of VEGF in podocytes (23). VEGF can deform and reduce the foot processes that make up the glomerular filtration membrane, resulting in proteinuria (23). Therefore, we examined the expression level of VEGF in the kidneys of mice. The expression level of VEGF in the kidneys of diabetic mice was increased, and the expression level of podocin was decreased. However, the expression level of VEGF in the kidneys of diabetic mice treated with 3-MA decreased, and the expression level of podocin increased. These results indicate that 3-MA improves the filtration barrier function in diabetic mice, which is confirmed by the results of 24-hour albuminuria.

DN is the most common cause of ESRD and the leading cause of glomerulosclerosis (1). DN is pathologically characterized by glomerular hypertrophy, and massive deposition of ECM in the tubules and glomeruli leading to tubular fibrosis and glomerulosclerosis (36, 37). The kidney is highly susceptible to hyperglycemia which causes kidney damage through multiple cellular pathways, including genetic activation and expression, increased production of AGEs, and increased oxidative stress, resulting in the activation of growth factors, transcription factors, and other molecules (38, 39). Growth factor and transcription factor regulatory genes encode some cytokines (such as IL-1, TNF-α, VEGF, etc.) which are involved in inflammation and ECM synthesis, and then cause renal inflammation, fibrosis, epithelial-mesenchymal transition (EMT), and dysfunction of parenchymal cells (such as endothelial cells, podocytes, renal tubular epithelial cells, etc.)(30, 40, 41). In this experiment, we first observed the general appearance of the kidney in diabetic mice, which showed paleness, edema, and hypertrophy, the upper and lower edges of the kidney were blunt and rounded, and the curvature of the left and right edges disappeared. However, the general appearance of kidneys in diabetic mice treated with 3-MA was significantly improved. Moreover, 3-MA treatment decreased the kidney/body weight ratio and glomerular volume significantly. PAS staining further showed that 3-MA treatment improved the pathology of glomerular hypertrophy, mesangial expansion, and tubular dilation. These data indicate that 3-MA ameliorates renal hypertrophy and injury in diabetic mice.

To further evaluate the beneficial effects of 3-MA on the kidneys of diabetic mice, we examined inflammation- and fibrosis-related biomarkers using ELISA, western blot, histology, and IHC staining. Both ELISA and western blotting results showed that 3-MA significantly lowered the expression levels of IL-1β and TNF-α in the kidneys of diabetic mice, which indicate that 3-MA improved the inflammatory response in the kidneys of diabetic mice. Western blotting results also showed that 3-MA remarkably lowered the expression levels of fibronectin and α-SMA which are related biomarkers of renal fibrosis in diabetic mice, indicating that 3-MA improved renal ECM deposition in diabetic mice. To further verify the improvement effect of 3-MA on renal ECM deposition in diabetic mice, we performed Sirius Red and IHC staining to observe the deposition of renal interstitial collagen fibers. Sirius Red staining showed that the kidneys of diabetic mice suffered serious morphological damage, including mesangial expansion, interstitial expansion, a large number of pink collagen deposition, and an increase in the positive area of Sirius Red staining. 3-MA treatment significantly improved the above symptoms. Fibronectin IHC staining also indicated that 3-MA lowered the increased fibronectin expression in the kidneys of diabetic mice. This robust evidence suggests that 3-MA significantly improves renal injury in diabetic mice.

Autophagy is a protective intracellular process (7). Recently, it has been reported that 3-MA improves hyperuricemia nephropathy and acute lung injury by inhibiting autophagy (14, 18, 19). To study the latent role of autophagy in the pathogenesis of DN, the expression levels of autophagy-related biomarkers were examined. We found that the ratio of LC3 II/LC3 I and the expression levels of Beclin-1 in the kidneys of diabetic mice were significantly increased. Since LC3 II/LC3 I and Beclin-1 are the biomarkers for the induction of autophagy (18), it is suggested that hyperglycemia induces renal autophagy. Interestingly, we also found that the expression level of P62 was also significantly raised in the kidneys of diabetic mice. P62 is a biomarker reflecting autophagic flux, and its increased level indicates that autophagic flux is blocked (42). The above results mean that hyperglycemia induces autophagy, but hinders autophagic flux. A large number of autophagosomes accumulating in the cell will affect the turnover of substances and may interact with apoptosis-related proteins to increase the cell burden (43-45). Therefore, induction of functionally impaired autophagy may make matters worse. 3-MA is an inhibitor of early autophagosome formation, which inhibits the formation of autophagosomes by inhibiting PI3K, thereby reducing the adverse consequences of the accumulation of autophagosomes (10). In this research, we discovered that 3-MA inhibited autophagy and lowered the accumulation of autophagosomes in the kidneys of diabetic mice, which is consistent with the findings of Bao *et al*. in hyperuricemia nephropathy (18). This evidence suggests that 3-MA may play a protective role in the kidneys of diabetic mice by inhibiting autophagosome formation.

HMGB1 is a DNA-binding protein that is highly conserved during eukaryotic evolution, it can regulate the stability of nucleosome structure and affect the stability of the binding of transcription factors to target gene sequences (46-48). Recently, HMGB1 was found to be a potent pro-inflammatory cytokine during infection (49). HMGB1 is a recognized alarming of inflammatory response, which is not only passively released by necrotic cells, but also autonomously secreted by immune cells, and is involved in the pathogenesis of various inflammatory responses (47, 50). HMGB1 contains two domains, A-box and B-box, and its B-box can act as an endogenous damage-associated molecular pattern (DAMP) to bind to TLR4 and activate the downstream transcription factor NF-κB, and thus exert its pro-inflammatory and pro-fibrotic effects(11, 33, 34, 51). In the present study, we found that the expression level of HMGB1 in the kidneys of diabetic mice was significantly up-regulated and the downstream NF-κB was activated. However, 3-MA inhibited the up-regulation of HMGB1 in the kidneys of diabetic mice, which in turn inhibited the activation of NF-κB. This evidence suggests that 3-MA may ameliorate renal inflammation and fibrosis in diabetic mice by inhibiting the activation of the HMGB1/NF-κB signaling pathway. 

## Conclusion

Our study demonstrates that the autophagy inhibitor 3-MA can attenuate renal injury in STZ-induced diabetic mice through multiple pathways. 3-MA ameliorates renal inflammation, ECM deposition, hypertrophy, and filtration barrier function in STZ-induced diabetic mice by reducing blood glucose, inhibiting autophagy, and inhibiting the activation of the HMGB1/NF-κB signaling pathway. 

## Authors’ Contributions

HW R and BJ L conceived and designed the experiments; MX H, LW O, X D, and X W contributed to the acquisition of data or analysis of data; HW R and BJ L drafted the manuscript; Q G and BJ L supervised the study. All authors read and approved the final version to be published.

## Ethical Statement

Human subjects are not involved in this study. This research protocol was approved by the Animal Ethics Committee of Changjiang University (Jingzhou, China, approval number: CJYXBEC2019-083), complies with the provisions of the Helsinki Declaration (revised in Edinburgh in 2000), and strictly complies with the “Guidelines for the Care and Use of Experimental Animals” (National Institutes of Health, Bethesda, Maryland, United States).

## Conflicts of Interest

The authors report no conflicts or interest in this work.

## References

[1] Mallipattu SK, He JC (2016). The podocyte as a direct target for treatment of glomerular disease?. Am J Physiol Renal Physiol.

[2] Shemesh II, Rozen-Zvi B, Kalechman Y, Gafter U, Sredni B (2014). AS101 prevents diabetic nephropathy progression and mesangial cell dysfunction: Regulation of the AKT downstream pathway. PLoS One.

[3] Tung CW, Hsu YC, Shih YH, Chang PJ, Lin CL (2018). Glomerular mesangial cell and podocyte injuries in diabetic nephropathy. Nephrology.

[4] Ma J, Chadban SJ, Zhao CY, Chen X, Kwan T, Panchapakesan U (2014). TLR4 activation promotes podocyte injury and interstitial fibrosis in diabetic nephropathy. PLoS One.

[5] Lin M, Yiu WH, Wu HJ, Chan LYY, Leung JCK, Au WS (2012). Toll-like receptor 4 promotes tubular inflammation in diabetic nephropathy. J Am Soc Nephrol.

[6] Ma J, Wu H, Zhao CY, Panchapakesan U, Pollock C, Chadban SJ (2014). Requirement for TLR2 in the development of albuminuria, inflammation and fibrosis in experimental diabetic nephropathy. Int J Clin Exp Pathol.

[7] Dikic I, Elazar Z (2018). Mechanism and medical implications of mammalian autophagy. Nat Rev Mol Cell Biol.

[8] Klionsky DJ, Abdel-Aziz AK, Abdelfatah S, Abdellatif M, Abdoli A, Abel S (2021). Guidelines for the use and interpretation of assays for monitoring autophagy (4th edition)1. Autophagy.

[9] Parzych KR, Klionsky DJ (2014). An overview of autophagy: Morphology, mechanism, and regulation. Antioxid Redox Signal.

[10] Bo Q, Shen M, Xiao M, Liang J, Zhai Y, Zhu H (2020). 3-Methyladenine alleviates experimental subretinal fibrosis by inhibiting macrophages and M2 polarization through the PI3K/Akt pathway. J Ocul Pharmacol Ther.

[11] Zhao Z, Hu Z, Zeng R, Yao Y (2020). HMGB1 in kidney diseases. Life Sci.

[12] Guo Y, Xiao Z, Wang Y, Yao W, Liao S, Yu B (2018). Sodium butyrate ameliorates streptozotocin-induced type 1 diabetes in mice by inhibiting the HMGB1 expression. Front Endocrinol (Lausanne).

[13] Wang B, Yang H, Fan Y, Yang Y, Cao W, Jia Y (2017). 3-Methyladenine ameliorates liver fibrosis through autophagy regulated by the NF-κB signaling pathways on hepatic stellate cell. Oncotarget.

[14] Ding D, Xu S, Zhang H, Zhao W, Zhang X, Jiang Y (2018). 3-Methyladenine and dexmedetomidine reverse lipopolysaccharide-induced acute lung injury through the inhibition of inflammation and autophagy. Exp Ther Med.

[15] Jung J, Choi H, Son E-D, Kim H (2021). 3-Methyladenine inhibits Procollagen-1 and fibronectin expression in dermal fibroblasts independent of autophagy. Curr Mol Med.

[16] Zou M, Wang F, Gao R, Wu J, Ou Y, Chen X (2016). Autophagy inhibition of hsa-miR-19a-3p/19b-3p by targeting TGF-β R II during TGF-β1-induced fibrogenesis in human cardiac fibroblasts. Sci Rep.

[17] He S, Zhou Q, Luo B, Chen B, Li L, Yan F (2020). Chloroquine and 3-Methyladenine attenuates periodontal inflammation and bone loss in experimental periodontitis. Inflammation.

[18] Bao J, Shi Y, Tao M, Liu N, Zhuang S, Yuan W (2018). Pharmacological inhibition of autophagy by 3-MA attenuates hyperuricemic nephropathy. Clin Sci (Lond).

[19] Shi Y, Tao M, Ma X, Hu Y, Huang G, Qiu A (2020). Delayed treatment with an autophagy inhibitor 3-MA alleviates the progression of hyperuricemic nephropathy. Cell Death Dis.

[20] Ren H-W, Yu W, Wang Y-N, Zhang X-Y, Song S-Q, Gong S-Y (2023). Effects of autophagy inhibitor 3-methyladenine on a diabetic mice model. Int J Ophthalmol.

[21] Liu B, He X, Li S, Xu B, Birnbaumer L, Liao Y (2017). Deletion of diacylglycerol-responsive TRPC genes attenuates diabetic nephropathy by inhibiting activation of the TGFβ1 signaling pathway. Am J Transl Res.

[22] Shanak S, Saad B, Zaid H (2019). Metabolic and epigenetic action mechanisms of antidiabetic medicinal plants. Evid Based Complement Alternat Med.

[23] Tufro A, Veron D (2012). VEGF and podocytes in diabetic nephropathy. Semin Nephrol.

[24] Benetti A, Martins FL, Sene LB, Shimizu MHM, Seguro AC, Luchi WM (2021). Urinary DPP4 correlates with renal dysfunction, and DPP4 inhibition protects against the reduction in megalin and podocin expression in experimental CKD. Am J Physiol Renal Physiol.

[25] Tang G, Li S, Zhang C, Chen H, Wang N, Feng Y (2021). Clinical efficacies, underlying mechanisms and molecular targets of Chinese medicines for diabetic nephropathy treatment and management. Acta Pharm Sin B.

[26] Ma T-T, Meng X-M (2019). TGF-β/Smad and renal fibrosis. Adv Exp Med Biol.

[27] Xu B-H, Sheng J, You Y-K, Huang X-R, Ma RCW, Wang Q (2020). Deletion of Smad3 prevents renal fibrosis and inflammation in type 2 diabetic nephropathy. Metabolism.

[28] Calle P, Hotter G (2020). Macrophage phenotype and fibrosis in diabetic nephropathy. Int J Mol Sci.

[29] Rayego-Mateos S, Morgado-Pascual JL, Opazo-Ríos L, Guerrero-Hue M, García-Caballero C, Vázquez-Carballo C (2020). Pathogenic pathways and therapeutic approaches targeting inflammation in diabetic nephropathy. Int J Mol Sci.

[30] Wada J, Makino H (2013). Inflammation and the pathogenesis of diabetic nephropathy. Clin Sci (Lond).

[31] Ashrafi Jigheh Z, Ghorbani Haghjo A, Argani H, Roshangar L, Rashtchizadeh N, Sanajou D (2019). Empagliflozin alleviates renal inflammation and oxidative stress in streptozotocin-induced diabetic rats partly by repressing HMGB1-TLR4 receptor axis. Iran J Basic Med Sci.

[32] Wu H, Chen Z, Xie J, Kang L-N, Wang L, Xu B (2016). High mobility group Box-1: A missing link between diabetes and its complications. Mediators Inflamm.

[33] Chen X, Ma J, Kwan T, Stribos EGD, Messchendorp AL, Loh YW (2018). Blockade of HMGB1 attenuates diabetic nephropathy in mice. Sci Rep.

[34] Zhang Y (2021). MiR-92d-3p suppresses the progression of diabetic nephropathy renal fibrosis by inhibiting the C3/HMGB1/TGF-β1 pathway. Biosci Rep.

[35] Thomas MC, Brownlee M, Susztak K, Sharma K, Jandeleit-Dahm KAM, Zoungas S (2015). Diabetic kidney disease. Nat Rev Dis Primers.

[36] Stefan G, Stancu S, Zugravu A, Petre N, Mandache E, Mircescu G (2019). Histologic predictors of renal outcome in diabetic nephropathy: Beyond renal pathology society classification. Medicine (Baltimore).

[37] Lei L, Mao Y, Meng D, Zhang X, Cui L, Huo Y (2014). Percentage of circulating CD8+ T lymphocytes is associated with albuminuria in type 2 diabetes mellitus. Exp Clin Endocrinol Diabetes.

[38] El Mesallamy HO, Ahmed HH, Bassyouni AA, Ahmed AS (2012). Clinical significance of inflammatory and fibrogenic cytokines in diabetic nephropathy. Clin Biochem.

[39] Sanchez AP, Sharma K (2009). Transcription factors in the pathogenesis of diabetic nephropathy. Expert Rev Mol Med.

[40] Pérez-Morales RE, Del Pino MD, Valdivielso JM, Ortiz A, Mora-Fernández C, Navarro-González JF (2019). Inflammation in diabetic kidney disease. Nephron.

[41] Lee HS (2012). Paracrine role for TGF-β-induced CTGF and VEGF in mesangial matrix expansion in progressive glomerular disease. Histol Histopathol.

[42] Au AK, Aneja RK, Bayır H, Bell MJ, Janesko-Feldman K, Kochanek PM (2017). Autophagy biomarkers Beclin 1 and p62 are increased in cerebrospinal fluid after traumatic brain injury. Neurocrit Care.

[43] Rubinstein AD, Kimchi A (2012). Life in the balance - a mechanistic view of the crosstalk between autophagy and apoptosis. J Cell Sci.

[44] Maejima Y, Isobe M, Sadoshima J (2016). Regulation of autophagy by Beclin 1 in the heart. J Mol Cell Cardiol.

[45] Mariño G, Niso-Santano M, Baehrecke EH, Kroemer G (2014). Self-consumption: The interplay of autophagy and apoptosis. Nat Rev Mol Cell Biol.

[46] Cheng M, Liu H, Zhang D, Liu Y, Wang C, Liu F (2015). HMGB1 enhances the AGE-Induced expression of CTGF and TGF-β via RAGE-dependent signaling in renal tubular epithelial cells. Am J Nephrol.

[47] Chen H, Li N, Zhan X, Zheng T, Huang X, Chen Q (2021). Capsaicin protects against lipopolysaccharide-induced acute lung injury through the HMGB1/NF-κB and PI3K/AKT/mTOR Pathways. J Inflamm Res.

[48] Wang Y, Zhong J, Zhang X, Liu Z, Yang Y, Gong Q (2016). The role of HMGB1 in the pathogenesis of type 2 diabetes. J Diabetes Res.

[49] Lee W, Yuseok O, Yang S, Lee B-S, Lee J-H, Park EK (2019). JH-4 reduces HMGB1-mediated septic responses and improves survival rate in septic mice. J Cell Biochem.

[50] Personnaz J, Piccolo E, Branchereau M, Filliol A, Paccoud R, Moreau E (2019). Macrophage-derived HMGB1 is dispensable for tissue fibrogenesis. FASEB Bioadv.

[51] Cheng M, Liu H, Zhang D, Liu Y, Wang C, Liu F (2015). HMGB1 enhances the AGE-induced expression of CTGF and TGF-β via RAGE-dependent signaling in renal tubular epithelial cells. Am J Nephrol.

